# Supplementary material for: Soil solution in Swiss forest stands: A 20 year's time series
Source: PLoS One. 2020 Jul 14;15(7):e0227530. doi: 10.1371/journal.pone.0227530 (PMC7360040; doi:10.1371/journal.pone.0227530)
Supplement: S1 File — (PDF) [file pone.0227530.s001.pdf]

Supplementary information

Soil solution in Swiss forest stands: a 20 year's time series

Braun Sabine[1]*, Tresch Simon[1], Sabine Augustin[2]

1 Institute for Applied Plant Biology, Benkenstrasse 254A, 4108 Witterswil, Switzerland
2 Federal Office for the Environment, 3003 Bern, Switzerland

* sabine.braun@iap.ch

1 Supplementary Figures

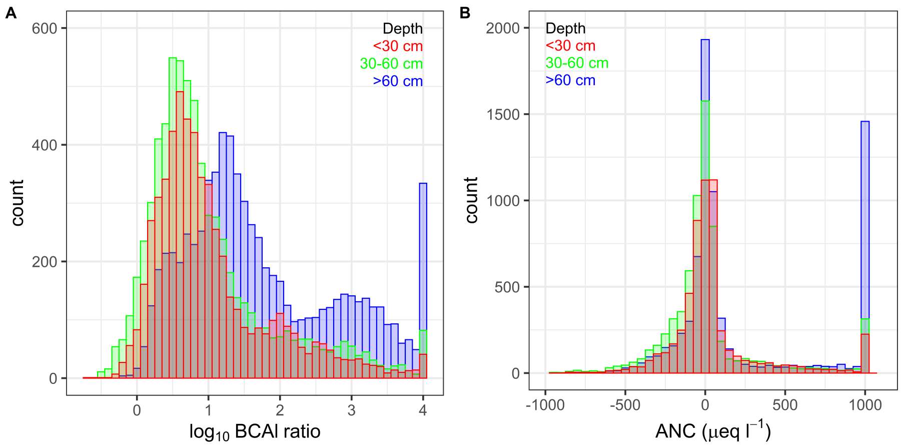

Figure S1. Density distribution of BC/Al (A) and ANC (B). Monthly samples. In the case of ANC, values of > 1000 were assigned to 1000 in order to make the values around zero more visible.

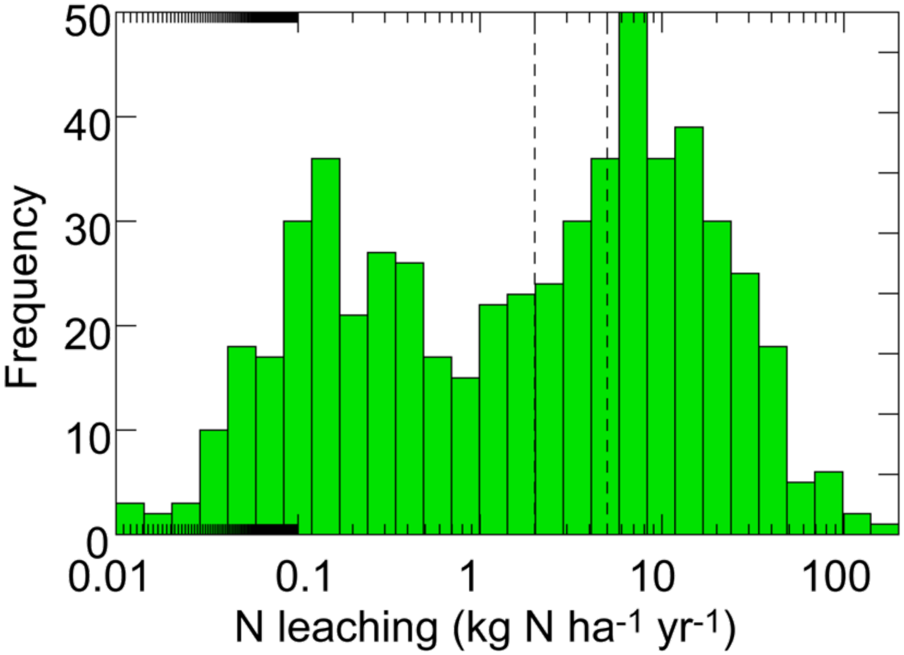

Figure S2. Annual rates of nitrogen leaching 2005-2016. The limits for coniferous and for deciduous trees (2 and 4 kg N ha^{-1} yr^{-1}) are indicated as dashed lines.

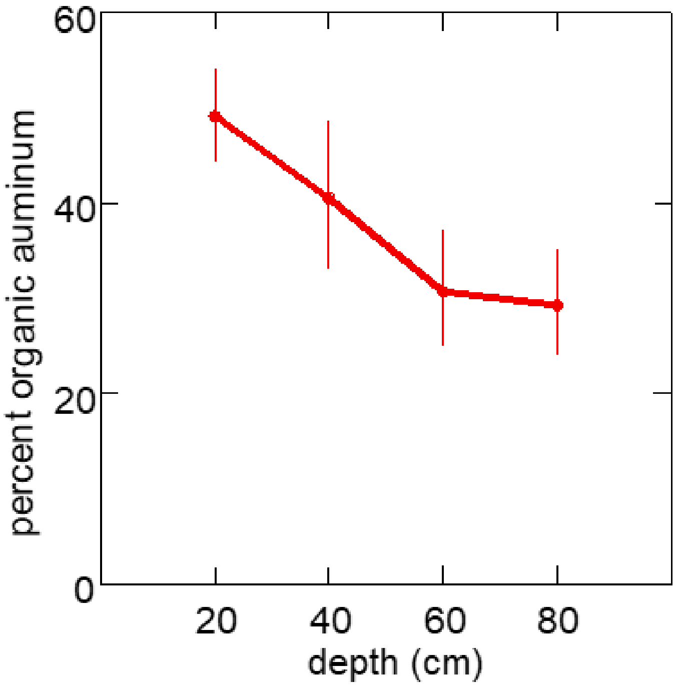

Figure S3. Proportion of organic complexed aluminum in relation to total aluminum for all samples since 2005 (n = 3204). Bars = 95 % confidence interval (mixed regression).

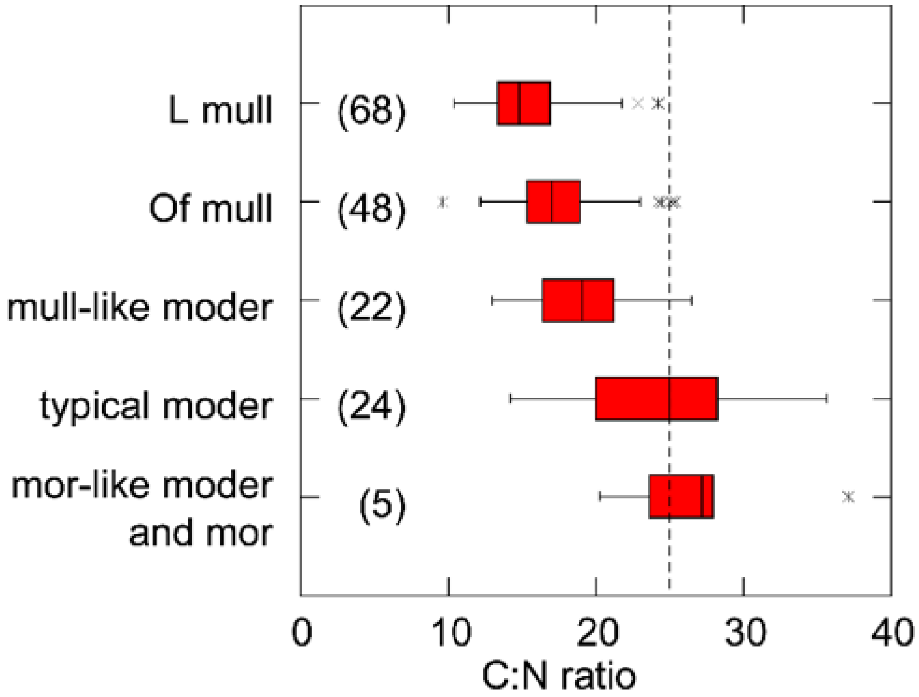

Figure S4. C:N ratio in all monitoring plots in relation to humus form. Dashed line: ratio of 25 (threshold to increased N leaching according to [1].

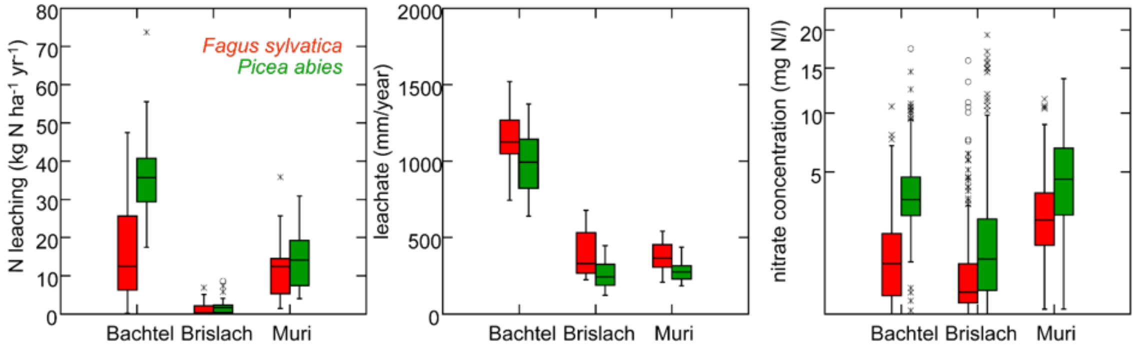

Figure S5. N leaching (left), amount of leaching water (middle) and nitrate concentration (right) in the soil solution of three plot pairs with neighbouring beech and Norway spruce. Data 2005 to 2016.

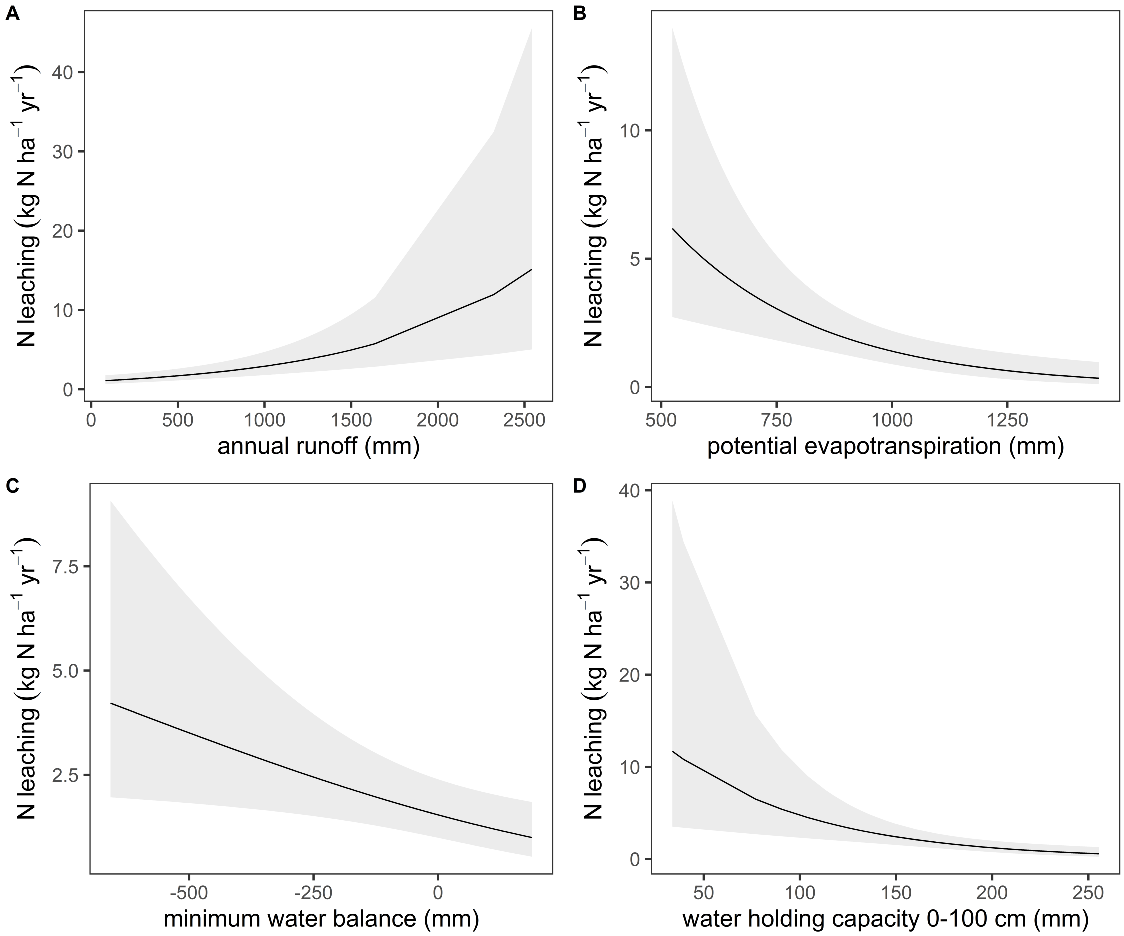

Figure S6. Relation between N leaching (kg N ha^{-1} yr^{-1}) and **A)** annual runoff, **B)** potential evapotranspiration, **C)** minimum water balance, **D)** water holding capacity, based on the linear mixed regression model given in Table 4.

2 Supplementary Tables

Table S1. Multiple linear regression model of the BC/AL ratio and base saturation with a total variance explained of Adj.$R^2 = 0.73$ **(A)**, as well as BC/AL ratio and pH($CaCl_2$) with a total variance explained of Adj.$R^2 = 0.55$ **(B)**.

A

	Coefficient	SE	t	P-value	
(Intercept)	0.546	0.069	7.920	< 0.001	***
base saturation	0.022	0.001	17.673	< 0.001	***

B

	Coefficient	SE	t	P-value	
(Intercept)	-10.3	2.06	-4.99	< 0.001	***
pH($CaCl_2$)	4.14	0.82	5.03	< 0.001	***
Second order orthogonal polynomial pH($CaCl_2$)	-0.32	0.08	-4.13	< 0.001	***

Table S2. Rates of leaching water, median values of nitrate concentration in different depths, nitrate leaching, modelled N deposition and evaluation according to the criteria of [2]. Average data for the years 2005-2018 (BUR, BE, GE, GI, GW, SE, ST, WSZ 2017 and 2018 only). In SA throughfall measurements suggested that the modelled N deposition is too low in this plot close to the Italian border (see also [3]).

Site	Leaching water	Nitrate concentration			N leaching	N deposition	Exc. critical limits	
		≤30 cm	>30-60cm	>60cm			conc.	leach.
	mm	mg N/l	mg N/l	mg N/l	kg N ha^{-1} a^{-1}	kg N ha^{-1} a^{-1}		
AW	482	2.34		1.73	8.1	25.7	x	x
AU	783	0.98	0.98	1.13	9.6	33.9	x	x
AI	472	3.36	1.99	2.25	6	33.4	x	x
AL	153		30.25		55.2	25.7	x	x
BAB	1093	1.29	1.22	0.22	14.1	30.3	x	x
BA	998	4.16	3.44	2.86	32.8	36.3	x	x
BE	321	6	8.64	4.83	12.8	60.9	x	x
BO	417	0.04		0.01	0.1	16.9		
BRAU	400	4.34	3.14	1.98	8.2	45.9	x	x
BB	346	0.09		0.03	0.1	22.1		
BRB	378	0.27	0.09		0.3	22.1	x	
BR	258	1.88	0.86	0.75	1.3	25.8	x	
BUR	572	1.58			11	32.8	x	x
BU	388	0.08		0.04	0.2	34.1		
DI	290	6.27	5.82	6.28	18.4	23.8	x	x
FR	542	0.35	1.02	1.62	4.4	27.8	x	x
GE	451	1.96	3.34	0.29	0.7	37.3	x	
GI	479	1.7			7.7	24.7	x	x
GB	961	0.94			6.8	22.5	x	x
GW	320	0.12	0.13	0.09	0.4	55.2		
HA	308	2.09		2.37	5.8	25	x	x
HI	619	2.03		1.92	12.1	36.3	x	x
LC	811	0.3	0.63	0.05	0.5	34.6	x	
LUB	1098			1.14	12.7	15.7	x	x
LU	1123	0.2	0.04	0.36	4.9	14.9		x
MO	267	4.8	3.97	2.33	5.8	23.3	x	x
MUB	340			3.01	11.8	32.5	x	x
MUF	278	4.55	4.41	4.2	12.5	37.6	x	x
MU	588	4.87	4.42	6.14	24.5	39.4	x	x
MUU	228		0.73	0.46	1.4	21.6	x	
OS	541	0.05	0.03	0.02	0.1	26.6		
OL	240	0.17	0.08	0.03	0.1	23.2		
PR	339	0.37		0.25	0.9	23.9	x	
RAF	315	1.02		0.06	0.2	23.6	x	
RI	402	0.84		1.47	2.8	19.7	x	
RU	245	0.06		0.09	0.3	23.5		
SA	943		6.74		52.3	36.1	x	x
SW	547	0.11	0.07	0.07	0.5	23.9		
SE	450	8.7	7.62	4.57	16.2	47.9	x	x
ST	924	7.43			44.9	32.5	x	x
WG	450	3.53	4.76	4.18	17.1	34.6	x	x
WSZ	634	0.66	0.32		0.4	31.1	x	
WA	922	0.04	0.05	0.07	0.6	12.7		
WI	465	1.08		0.72	3.4	28.9	x	
ZO	370	0.45		0.55	1.8	26.8	x	
ZBB	900	1.35	0.61	0.05	5.5	33	x	x
ZB	821	1.86	2.24	1.08	8	32.6	x	x
ZV	550	1.82		1.34	7.8	34.3	x	x
Average	538	2.0	3.4	1.5	9.4	30.0		
Number							38	28
Percent							79	58

Table S3. Linear mixed regression model of log transformed N leaching (kg N ha^{-1} yr $^{-1}$) and year with plots per year as a random effect. Pseudo-R^2 fixed effects = 0.02, Pseudo-R^2 including random effect = 0.74.

	Coefficient	SE	t	df	P-value	
(Intercept)	110.89	18.09	6.13	553.10	< 0.001	***
year	-0.05	0.01	-6.09	552.66	< 0.001	***

Table S4. Linear mixed regression model of N leaching (kg N ha^{-1} yr $^{-1}$). Dependent variable: N leaching in kg N ha^{-1} yr $^{-1}$ log transformed. The combined effect of annual tree removal rates (Figure 7 B) was calculated as weighted average based on the model coefficients of lag 0, 1, 2 and 3 (Table 4) and the combined effect of drought indicators was calculated as the weighted average of the model coefficients of leaching water, minimum site water balance and potential evapotranspiration (Table 4). Pseudo-R2 fixed effects = 0.37, Pseudo-R2 including random effect = 0.77, (n=586).

	Coefficient	SE	t	df	P-value	
(Intercept)	3.04	1.07	2.84	71.11	0.006	**
N deposition	0.10	0.02	4.95	75.64	< 0.001	***
annual tree removal (0-3 years)	6.59	1.08	6.09	535.94	< 0.001	***
drought indicators	-4.32	0.68	-6.35	212.44	< 0.001	***
water holding capacity	-0.01	0.00	-3.25	50.17	0.002	**

Table S5. Linear regression model of the log transformed Ca leaching and Ca input from weathering and deposition. Adj.R^2= 0.52, n=586.

	Coefficient	SE	t	P-value	
(Intercept)	0.22	0.19	1.18	0.245	
log(Ca input)	1.00	0.15	6.65	< 0.001	***

Table S6. Linear regression model of the log transformed Ca leaching in relation to weathering rate, Ca deposition and water holding capacity. N deposition and the proportion of coniferous trees were excluded based on the AIC of the model comparison. Adj.R^2= 0.56, n=41.

	Coefficient	SE	t	P-value	
(Intercept)	3.05	0.85	3.59	0.001	**
log(weathering rate)	0.55	0.12	4.71	< 0.001	***
log(Ca deposition)	1.42	1.14	1.24	0.223	
soil water holding capacity	-0.01	0.00	-2.32	0.026	*

Table S7. Linear mixed regression model of the BC/Al ratio with site number as random effect and year, soil depth and base saturation levels as a fixed effects. Pseudo-R^2 fixed effects = 0.53, Pseudo-R^2 including random effect = 0.76, n=17366.

A

	Coefficient	SE	t	df	P-value	
(Intercept)	38.37	1.48	26.01	16794.06	< 0.001	***
Year	-0.02	0.00	-25.89	17336.59	< 0.001	***
base saturation > 15-40 %	0.44	0.19	2.34	43.65	0.024	*
base saturation > 40 %	2.01	0.21	9.68	43.97	< 0.001	***
soil depth 30-60 cm	0.15	0.01	14.44	17337.23	< 0.001	***
soil depth > 60 cm	0.87	0.01	89.13	17333.61	< 0.001	***

Linear mixed regression model of the BC/Al ratio for soil depths < 30 cm with site number as random effect and year and base saturation levels as a fixed effects. Pseudo-R^2 fixed effects = 0.57, Pseudo-R^2 including random effect = 0.81, n=6608.

B

	Coefficient	SE	t	df	P-value	
(Intercept)	43.41	1.85	23.43	6603.53	< 0.001	***
Year	-0.02	0.00	-23.23	6579.55	< 0.001	***
base saturation > 15-40 %	0.35	0.17	2.04	40.64	0.047	*
base saturation > 40 %	1.84	0.18	10.06	40.94	< 0.001	***

Linear mixed regression model of the BC/Al ratio for soil depths 30 - 60 cm with site number as random effect and year and base saturation levels as a fixed effects. Pseudo-R^2 fixed effects = 0.40, Pseudo-R^2 including random effect = 0.72, n=5065.

C

	Coefficient	SE	t	df	P-value	
(Intercept)	32.27	2.33	13.87	5058.36	< 0.001	***
Year	-0.02	0.00	-13.69	5048.20	< 0.001	***
base saturation > 15-40 %	0.72	0.21	3.42	23.90	0.002	**
base saturation > 40 %	2.15	0.30	7.20	24.28	< 0.001	***

Linear mixed regression model of the BC/Al ratio for soil depths > 60 cm with site number as random effect and year and base saturation levels as a fixed effects. Pseudo-R^2 fixed effects = 0.40, Pseudo-R^2 including random effect = 0.77, n=5693.

D

	Coefficient	SE	t	df	P-value	
(Intercept)	30.82	2.70	11.44	5688.46	< 0.001	***
Year	-0.01	0.00	-11.03	5665.12	< 0.001	***
base saturation > 15-40 %	0.47	0.26	1.77	31.97	0.087	
base saturation > 40 %	1.96	0.30	6.54	32.13	< 0.001	***

Linear mixed regression model of the BC/Al ratio for soil depths < 30 cm and a base saturation < 15 % with site number as random effect and year as a fixed effects. Pseudo-R^2 fixed effects = 0.07, Pseudo-R^2 including random effect = 0.41, n=1908.

E

	Coefficient	SE	t	df	P-value	
(Intercept)	34.87	2.43	14.36	1904.51	< 0.001	***
Year	-0.02	0.00	-14.16	1903.22	< 0.001	***

Linear mixed regression model of the BC/Al ratio for soil depths < 30 cm and a base saturation of 15-40 % with site number as random effect and year as a fixed effects. Pseudo-R^2 fixed effects = 0.06, Pseudo-R^2 including random effect = 0.51, n=3225.

F

	Coefficient	SE	t	df	P-value	
(Intercept)	43.21	2.19	19.72	3217.37	< 0.001	***
Year	-0.02	0.00	-19.34	3212.35	< 0.001	***

Linear mixed regression model of the BC/Al ratio for soil depths < 30 cm and a base saturation > 40 % with site number as random effect and year as a fixed effects. Pseudo-R^2 fixed effects = 0.03, Pseudo-R^2 including random effect = 0.60, n=1475.

G

	Coefficient	SE	t	df	P-value	
(Intercept)	62.84	6.69	9.40	1469.56	< 0.001	***
Year	-0.03	0.00	-9.06	1468.26	< 0.001	***

Linear mixed regression model of the BC/Al ratio for soil depths 30-60 cm and a base saturation < 15 % with site number as random effect and year as a fixed effects. Pseudo-R^2 fixed effects = 0.05, Pseudo-R^2 including random effect = 0.57, n=1613.

H

	Coefficient	SE	t	df	P-value	
(Intercept)	31.96	2.39	13.37	1609.66	< 0.001	***
Year	-0.02	0.00	-13.17	1606.72	< 0.001	***

Linear mixed regression model of the BC/Al ratio for soil depths 30-60 cm and a base saturation of 15-40 % with site number as random effect and year as a fixed effects. Pseudo-R^2 fixed effects = 0.06, Pseudo-R^2 including random effect = 0.51, n=3225.

I

	Coefficient	SE	t	df	P-value	
(Intercept)	43.21	2.19	19.72	3217.37	< 0.001	***
Year	-0.02	0.00	-19.34	3212.35	< 0.001	***

Linear mixed regression model of the BC/Al ratio for soil depths 30-60 cm and a base saturation > 40 % with site number as random effect and year as a fixed effects. Pseudo-R^2 fixed effects = 0.03, Pseudo-R^2 including random effect = 0.54, n=362.

J

	Coefficient	SE	t	df	P-value	
(Intercept)	47.47	13.07	3.63	359.85	< 0.001	***
Year	-0.02	0.01	-3.43	359.74	< 0.001	***

Linear mixed regression model of the BC/Al ratio for soil depths >60 cm and a base saturation < 15 % with site number as random effect and year as a fixed effects. Pseudo-R^2 fixed effects = 0.02, Pseudo-R^2 including random effect = 0.68, n=1880.

K

	Coefficient	SE	t	df	P-value	
(Intercept)	40.83	3.89	10.51	1876.36	< 0.001	***
Year	-0.02	0.00	-10.22	1870.44	< 0.001	***

Linear mixed regression model of the BC/Al ratio for soil depths >60 cm and a base saturation of 15-40 % with site number as random effect and year as a fixed effects. Pseudo-R^2 fixed effects = 0.01, Pseudo-R^2 including random effect = 0.66, n=2750.

L

	Coefficient	SE	t	df	P-value	
(Intercept)	29.18	4.05	7.20	2745.20	< 0.001	***
Year	-0.01	0.00	-6.80	2735.85	< 0.001	***

Linear mixed regression model of the BC/Al ratio for soil depths >60 cm and a base saturation of > 40 % with site number as random effect and year as a fixed effects. Pseudo-R^2 fixed effects = 0.00, Pseudo-R^2 including random effect = 0.41, n=1063.

M

	Coefficient	SE	t	df	P-value	
(Intercept)	16.21	7.34	2.21	1059.59	0.03	*
Year	-0.01	0.00	-1.79	1059.14	0.07	

References

1. Gundersen P, Callesen I, de Vries W. Nitrate leaching in forest ecosystems is related to forest floor CN ratios. Environmental Pollution. 1998;102(1):403–407. doi:10.1016/S0269-7491(98)80060-2.

2. CLRTAP. Mapping Critical Loads for Ecosytems. Chapter V of Manual on methodologies and criteria for modelling and mapping critical loads and levels and air pollution effects, risks and trends. UNECE Convention on Long-range Transboundary Air Pollution.; 2017.

3. Thimonier A, Kosonen Z, Braun S, Rihm B, Schleppi P, Schmitt M, et al. Total deposition of nitrogen in Swiss forests: Comparison of assessment methods and evaluation of changes over two decades. Atmospheric Environment. 2019;198(April 2018):335–350. doi:10.1016/j.atmosenv.2018.10.051.
