# Supplementary material for: Soil solution in Swiss forest stands: A 20 year's time series
Source: PLoS One. 2020 Jul 14;15(7):e0227530. doi: 10.1371/journal.pone.0227530 (PMC7360040; doi:10.1371/journal.pone.0227530)
Supplement: Attachment — Submitted filename: reviewer_comments_rev1.docx [file pone.0227530.s003.docx]

Reviewer #1:

5. Review Comments to the Author

Please use the space provided to explain your answers to the questions above. You may also include additional comments for the author, including concerns about dual publication, research ethics, or publication ethics. (Please upload your review as an attachment if it exceeds 20,000 characters)

Reviewer #1: This is a highly relevant and thoroughly elaborated.
It is quite interesting to read that the soil solution monitoring has been continued for 20 years and the future ahead.

Reviewer #1: The technical description of the approach and the discussion are very well written. The quality of the text is high and it could be published as it is.

Reviewer #1: Nevertheless, there is always room for improvement:
1/ The introduction of the manuscript is reflecting the state of knowledge of 30 years ago. At that time there was the concern that acidification threatens European forests, and 20 years ago it was stated that N eutrophication is a major concern. There have been many papers since them that put these concerns into perspective. A good source of information of such reviews are the TAMM Reviews of Forest Ecology and Management. - I recommend to either state in the Introduction that the text is reflecting the knowledge when the monitoring programmes were initiated, or (preferred) to expand the Introduction by more recent references.

*Author: Thank you for your valuable comments. We changed now the wording in the first sentence to show that soil acidification has been an issue in the 1980's.*

*Lines 29-32: Since the 1980s it has been recognized that soil acidification due to anthropogenic input of sulfur and nitrogen compounds ([1]) poses a serious threat to forest health ([2]). An increase in soil acidification, related to atmospheric acid deposition, has been reported in many European countries such as Germany ([3]), Sweden ([4] or France ([5]).*

*However, the later sentences have already stated that mitigation measures have improved the situation. Therefore, it is necessary to refer to older literature as they are the basis of the Critical Loads for Acidity. I do, however, not agree with the conclusions of the proposed Tamm review by Högberg [6] that there is no N problem as they refer to Northern Ecosystems where total N deposition is much lower and the economy of forest growth and thus biomass production are much more important. In Switzerland, forest N eutrophication is a problem, not a fertilization measure.*

Reviewer #1: 2/ The methods section is quite complex. The author repeatedly refers to extensive manuals where the single steps are described. Presumingly, very few readers will look up these sources. -- Considering that supplementary material comes with the paper, it would be desirable to show the code that had been used. -- Also a statement on confidence in the invidual steps would be interesting. E.g. how well does SAFE reflect weathering in the field?

*Author: We have now added a description on base saturation determination as this is the part which the reader should know without looking up – is this conclusion correct? We have added the R scripts we used to the supplementary material and a remark on validation of SAFE calculations to the methods section.*

Reviewer #1: 3/ The full data sets are not available. - If this is the data policy of the institute, a statement on data ownership could be made.

Author: We have uploaded all R codes and a description of data manipulation and models including the raw data on the data base dryad:

Data Availability: The data and an R project including an RMarkdown file with all R codes, model outputs and diagnostic plots have been uploaded to Dryad: <https://doi.org/10.5061/dryad.2z34tmphm>.

Before acceptance of the paper the data are accessible via:

https://datadryad.org/stash/share/PjiHqj7uwJK13MBFbbbiRVeoO_DI1byCEPpHDD3bDms

Reviewer #2:

Reviewer #2: The author has evaluated the chemistry of soil solution at a number of Swiss forest sites with respect to their excedance of critical limits for acidity and nitrate. In addition some models were run to relate the critical limits to site, stand, atmospheric inputs and a number of other factors.

Reviewer #2: First off, the premise of the paper is not sound. Line 28 states ‘Soil acidification is a serious threat’. This is not the case. Soil acidification is a natural process of soil development in temperate climates where there is a precipitation surplus. The objectives are not clearly stated and the results presented e.g. Ca budgets do not correspond to the stated aim on Line 64-66. Line 66 states that soil solution chemistry will be related to forest health but this is not presented. No evidence is presented to indicate that forests in Switzerland are suffering from the effects of acidification from atmospheric deposition of S and N. Is there forest health data available? Foliar chemistry for example? Evidence of magnesium deficiency or yellowing of needles?

*Author: Thank you for your comments. According to your suggestions we changed the first sentences of the introduction:*

*Lines 29-34: Since the 1980s it has been recognized that soil acidification due to anthropogenic input of sulfur and nitrogen compounds ([1]) poses a serious threat to forest health ([2]). An increase in soil acidification, related to atmospheric acid deposition, has been reported in many European countries such as Germany ([3]), Sweden ([4] or France ([5]). Due to mitigation measures, the deposition of acidifying substances in Europe, in particular of sulfur compounds, has decreased in recent years, but eutrophication due to the deposition of reactive nitrogen compounds remains a critical issue ([7], [8]).*

*The reader can now understand that measures have been taken against acid deposition. It is correct that soil acidification is a natural process but the pH reached with the main natural acidifying substance HCO3- is +/- pH 4.8-5.0, depending on the partial pressure of the CO2. In the uppermost cm of the mineral soil the organic acids can acidify the soil somewhat more, but the organic acids cannot reach the subsoil, because they dissociate in the Bs-horizon of a soil, at the latest (due to the normal pH gradient in soils). In contrast, the acidification due to the input of the mobile anions SO_4_^2-^ and NO_3_^-^ is more “effective”; SO_4_^2-^ can be stored in the B horizon, but this is not stable, the SO_4_^2-^- desorbs if the S-input declines. In this case, the deeper soil horizons acidify. This was demonstrated in a lot of intensive monitoring plots. Nitrate cannot be stored in the soil. If it is not taken up by vegetation, the NO3- passes through the profile, together with a concomitant cation (BC or an acid cation like Mn or Al), diminishing the BS% in soils. We have added now a reference to [1] to highlight these processes.*

*The relations between soil acidification and forest health have been discussed in the submitted manuscript on line 50 ([9]) and line 416 ([10] ). These two references show results from the forest plots presented in this study but we agree that this has not been stated clearly. We have now added a separate paragraph to the Discussion section including information on Mg deficiency which has also increased in recent years, and that foliar Mg has been related to soil solution Mg.*

*Lines 410-416: Effects of soil acidification on forest health in the forest plots presented in the current study have been observed on rooting depth [9], uprooting of trees [10] and on Mg deficiency [11]. The uprooting of trees was clearly increased on soils with a base saturation <40%. Visible Mg deficiency also has strongly increased within the last 10-15 years, suggesting that soil acidification is still an issue in the Intercantonal forest observation plots. Foliar Mg concentrations in beech leaves have been shown to be related with Mg concentrations in soil solution [11].*

Reviewer #2: The title of the paper is 'Soil solution in Swiss forest stands: a 20 year's time series' but no time series are presented.

*Author: This is not true. Figure 4 shows a time series of the BC/Al ratio and Figure 6 on N leaching. But with 47 plots it is not feasible to show all plots separately. The graphs for all single plots are, however, accessible through the project report ([12] ).*

Reviewer #2: It is not clear what the purpose of the N assessment is and how it relates to acidification and what it contributes to the paper. The author states that ‘N leaching may not always be a good eutrophication indicator’ [L22-23]. This has already been well established in the literature
e.g. Lovett, G.M., Goodale, C.L. A New Conceptual Model of Nitrogen Saturation Based on Experimental Nitrogen Addition to an Oak Forest. Ecosystems 14, 615–631 (2011). https://doi.org/10.1007/s10021-011-9432-z

*Author: The reason why N is related with acidification is that the negatively charged nitrate carries along positively charged ions. We have added a remark to the corresponding paragraph. We have also introduced a reference to Lovett & Goodale but their conclusion is not that N leaching is not a eutrophication indicator but that it may occur simultaneously with other stages of N saturation. The sentence that "N leaching may not always be a good eutrophication indicator" is in the abstract and cannot refer to a reference at this place.*

Reviewer #2: A lot of the literature cited is quite old e.g. refs 11, 16, 18, 19, 25, 27, 30. A lot of new knowledge has been published in relation to the issue of soil acidification by atmospheric deposition that the author should review.

*Author: We introduced a number of newer references ([8], [13], [14],[15], [16] ). However, when referring to the Critical Load manuals it is necessary to cite the old literature which was the basis to setting the limits.*

Reviewer #2: In addition, a lot of work done on Swiss forest monitoring specifically with respect to this issue, but this work is not cited. I would encourage the author to review these papers and position their work within this context. Sample below

Pannatier, E.G., Thimonier, A., Schmitt, M. et al. A decade of monitoring at Swiss Long-Term Forest Ecosystem Research (LWF) sites: can we observe trends in atmospheric acid deposition and in soil solution acidity?. Environ Monit Assess 174, 3–30 (2011). https://doi.org/10.1007/s10661-010-1754-3
Graf Pannatier, E., Walthert, L. and Blaser, P. (2004), Solution chemistry in acid forest soils: Are the BC : Al ratios as critical as expected in Switzerland?. Z. Pflanzenernähr. Bodenk., 167: 160-168. doi:10.1002/jpln.200321281
Thimonier, A., Graf Pannatier, E., Schmitt, M., Waldner, P., Walthert, L., Schleppi, P., et al. (2010a). Does exceeding the critical loads for nitrogen alter nitrate leaching, the nutrient status of trees and their crown condition at Swiss Long-term Forest Ecosystem Research (LWF) sites? European Journal of Forest Research, 129, 443–461.
Waldner, P., Schaub, M., Graf Pannatier, E., Schmitt, M., Thimonier, A., & Walthert, L. (2007). Atmospheric deposition and ozone levels in Swiss Forests: Are critical values exceeded? Environmental Monitoring and Assessment, 128, 5–17.

*Author: We have added the Pannatier 2011 reference..*

Reviewer #2: I question many aspects of the analysis.
Is Bc:Al expressed as mols of charge here? Is aluminium total Al? What is the basis that an exceedance >1% of the observations [Line 156] is biologically significant?
In calcareous soils, the Al concentrations will be negligible, which makes the Bc:Al ratio very large. This has a spurious effect on the statistical analysis e.g. Figure 2 has Bc:Al values up to 10,000. In the same figure Bc:Al for soil depth >70cm is shown, but Bc:Al is an indicator for stress on fine plant root yet the author states that root depth only extended to 60cm [L173]. So I don’t think Bc:Al below this depth is relevant. For a study of acidification, the analysis should be confined to those soils in the Al buffering pH range.

*Author: The calculation of BC/Al is described in the UNECE manuals but we have now inserted an explanation how we did it. The 1% limit was used to remove outliers. The mapping manual does not define how many values have to exceed the threshold but the roots will react to single values rather than any average. We agree that calculation of BC/Al in calcareous soils is subjected to errors but omitting these values would bias the dataset towards acid soils. Looking at outliers in the graphs is misleading as we have log transformed them before data analysis. We agree that BC/Al at soil depth >70 cm is not relevant for plant roots but we am showing these values for the sake of completeness, to show the trends in soil solution chemistry. We do not draw any plant relevant conclusions on these values.*

*To confine acidification studies only on soils with pH 3.8 – 4.2 (Al buffer range) would lead to wrong conclusions; to detect trends one should use the whole existing pH-range. Thus, we do not agree with the recommendation that the data analysis should be restricted to the Al buffer range.*

Reviewer #2: The regression analysis is not appropriate. It is not clear what the objective of this analysis is and many of the relationships have already been established in the literature. For example, why is Bsat a predictor for Bc:Al in soil solution? Bsat should be considered a response variable and not a predictor in this case. Also Bsat to Bc:Al relationships have already been developed and are used in the critical loads modelling e.g. Gaines-Thomas and Gapon equations.

*Author: The aim of the regression analysis was to find out why the decrease in BC/Al is going on fast in some plots and slow in others. We have added now an introducing sentence. The Gaines-Thomas and Gapon equations are useful in soil chemical models to describe the exchange of cations between soil solution and soil solid phase. Here we follow a statistical approach to explain the measured changes in the composition of the soil solution. For comparisons of the critical thresholds of BC/Al in soil solution with the base saturation of the soil solid phase it is important to have field data for validation*

Reviewer #2: I would also question the data used as an input to the model. The author states that the number of sites ranges from ‘9 to 47’. I don’t think it is appropriate to have uneven numbers of observations for different sites. Also it is not clear how the 5 year time interval [L186] was generated for predictors. Was soil Bsat, Soil C:N measured every 5 years? Was the weathering rate recalculated for every 5 year interval? Time intervals for a particular site are not independent observations – was this accounted for in the model?

*Author: The mixed regression accounts for unequal number of observation and for unequal replication. So from the statistical point of view it is not a problem to compare Bsat and C:N measured once or weathering rate modelled once with monthly measured soil solution data. The mixed regression handles also repeated measures from one site, e.g. time intervals.*

*The Bsat and soil C:N were not measured every 5 year, because it is not expectable to find measurable changes in such a short time, esp. in a heterogeneous environment like forest soils. The weathering rate is stable, it is enough to measure it once. In contrast, the composition of the soil solution is a fast reacting monitor of changes, that’s why it is measures monthly, and for the annual changes aggregated to annual means.*

Reviewer #2: The relationship presented in Figure 2, between pHCaCl2 and Bc:Al is spurious.

*Author: Figure 2 shows measured BC/Al ratio in relation to pH and base saturation. Based on this comment it is not clear how this is wrong or how it could be changed or improved.*

Reviewer #2: I don’t think the results presented in Table 3 are valid. The levels of significance are likely a result of the large number of observations used.

*Author: Figure 5 illustrates the regressions with the confidence intervals and the data points so the reader can check that the regressions are quite good and not just an artefact of the high number of points.*

Reviewer #2: If N leaching has decreased and N deposition is the main driver of acidity [L18-19] then why is the rate of soil acidification increasing [L17-18, L264]? Why wasn’t N deposition significant in the model of soil acidification (Table 3)?

*Author: The processes leading to soil acidification are not linear so a decrease in N deposition (which is actually small anyway in Switzerland) will not necessarily lead to an immediate decrease in soil acidification (for recovery effects see e.g. Verstraeten 2017). N is still accumulating as long as the deposition of N is larger than the use by the forest stand. The relation between the amount of N deposition and soil acidification can be shown in the experiment but in the field the geological heterogeneity is very large. We have now made a reference to this experiment.*

Reviewer #2: In Fig 3 the ca leaching rate is greater than deposition (and weathering + deposition. What is the proposed driver of this Ca loss, if N deposition is decreasing? How do these values relate to other published values for weathering? Was the weathering model calibrated? It has previously been reported that soil solution in acid pseudogleyed horizons can be influenced by underlying clay soils (Graf Pannatier 2004). Was this taken into account in the analysis?

*Author: The deposition in the X-axis is Ca-deposition, not N deposition. Thus, Figure 3 does not show N deposition but the Ca leaching is the result of NO3‑ -leaching.*

*The weathering model was calibrated in Sweden. Recent analyses with Al in tree rings confirm that the dynamic soil acidification model is performing quite well. We have put this remark to the methods section. – The indicated reference discusses the effect of underlying clay soils on soil solution in 80 cm depth. As the soil solution chemistry at larger depths is not in the focus of the current paper, such processes do not change the main conclusions. Similar situations (soil wetness >2 and calcareous layer present in <100 cm with acid topsoil) occur in just one among 47 plots.*

Reviewer #2: In relation to Fig. 5 it is not clear what this figure is trying to show. Why not simply plot Bc:Al change with time? Again the linear relationships don’t seem valid.

*Author: A plot of BC/Al change with time is given in Figure 4. Figure 5 illustrates the speed of the change in BC/Al in relation to the predictors in Table 3. Predicted values including 95% confidence intervals are conditioned on all other fixed effects. Negative changes signify an expected decrease in BC/Al. The linearity of the relations was tested using polynomial functions*

Reviewer #2: The statistical relationships in figures 6 to 8 don’t seem valid, but seem to be driven by a few outliers. Why does N leaching extend above 150KgN?

*Author: The regressions were made with log transformed values (this is stated in the text) in order to meet the assumptions for a liner mixed effect model. The models were tested for robustness. The shaded areas give the 95% confidence interval. For the graphs, the values were backtransformed so the scatter seems larger than it actually was.*

*The mixed regression model given in Table 4 explains about 77 % of the variation of N leaching. The amount of n=586 seems valid to test the given amount of fixed effects. These fixed effects explained alone 36% of the data. The Pseudo-R2 are now given in the tables.*

*E.g. Lines 370-372:*

*Table 4: Mixed regression model of N leaching with annual data (n=586). Dependent variable: N leaching in kg N ha 1 yr 1, log transformed. Pseudo-R2 fixed effects = 0.37, Pseudo-R2 including random effect = 0.77.*

*All models can now be seen in the Supplementary Materials in the RMarkdown documented R codes in order to achieve the highest standards of statistical reproductivity of the models.*

*The points with very high N leaching have been plot with disturbances. The points show annual leaching rates.*

Reviewer #2: The English needs to be improved and there are numerous grammatical errors and formatting errors. This makes the paper difficult to read. some examples.

*Author: A great effort has been invested to improve wordings and eliminate all grammatical errors.*

L374 comma at the end of line

*Author: Thank you for this note this has been changed.*

L13; ‘ranging’ instead of ‘growing’

*Author: Thank you for this comment: 'ranging' means that they extend from .. to. 'Growing' means that the number has increased. We have now replaced "growing" by "increasing".*

L18: ‘Main driver’ – should be ‘The main driver..’

*Author: Thank you for this note this has been changed.*
L36 'concerns on forest health'..should be 'concerns about forest health'

*Author: Thank you for this note this has been changed.*

The conclusions are not supported by the findings.

*Author: We cannot comment this as this is a statement without proof.*

6. PLOS authors have the option to publish the peer review history of their article ([what does this mean?](https://journals.plos.org/plosone/s/editorial-and-peer-review-process#loc-peer-review-history)). If published, this will include your full peer review and any attached files.


If you choose “no”, your identity will remain anonymous but your review may still be made public.


**Do you want your identity to be public for this peer review?** For information about this choice, including consent withdrawal, please see our [Privacy Policy](https://www.plos.org/privacy-policy).

Reviewer #1: Yes: Robert Jandl

Reviewer #2: No


[NOTE: If reviewer comments were submitted as an attachment file, they will be attached to this email and accessible via the submission site. Please log into your account, locate the manuscript record, and check for the action link "View Attachments". If this link does not appear, there are no attachment files to be viewed.]

*Additional changes:*

- *Figure 3: Point size increased and labels more close to points, Figure label A and B introduced.*

*Figure 3: Relation between the leaching of Ca at a depth of 60-80 cm (average 2005-2018) and the weathering rate of base cations cumulated over the uppermost 60 cm. A: Relation between Ca leaching and weathering rate. B: Relation between Ca leaching and the sum of Ca weathering and Ca deposition (model [17], for Southern Switzerland [18]). Site abbreviations for sites can be found in Table 1. Only sites with a high Ca leaching are labelled in the plot. The line represents the 1:1 line.*

- *Figure 2: New colours, better visability of regression equation and R2. Figure legend has been modified.*

*Figure 2: Relationship between the BC/Al ratio of the soil solution and base saturation (A) and between the BC/Al ratio and soil pH (B) . Points represents measurements from the soil layer below 70 cm depth. Triangles represents measurements form the soil layer above 70 cm depth.*

*Lines 91-94: Wording has changed:*

*The studied sites are part of the Intercantonal Forest Observation Program in Switzerland ([19]). The first suction cups were installed in nine plots of the total 189 plots in 1997, with samples being collected since 1998. More plots were added in subsequent years to give a current total of 47 plots with soil solution measurements (Table 1).*

*Lines 254-257 has been modified:*

*The exceedance of acidity limits according to the Geneva Air Convention ([20]) or suggested by [21] are listed in Table 2. The BC/Al ratio lower than 1 was observed in 27% of the plots (in at least one layer) and a BC/Al ratio lower than 7 was observed in 71% of the plots.*

*Figure 7 and 8 are now taken together:*

*Figure 7: A: Relationship between N leaching and N deposition (Table 4). B: Relationship between N leaching and tree removal averaged over 0-3 years (Table S4). Tree removal is in fraction of 1, i.e. 0.4 means that 40% of the trees are removed.*

*Figure 6 has been rearranged and replotted:*

*Figure 6: Development of N leaching 1998-2018. The decrease with time is significant with p<0.001. Thick squares and error bars (95% confidence intervals) are estimates corrected for the varying number of plots per year from mixed regression (Table S3). Small dots are raw measurements, n=586.*

*Figure 4 has been changed:*

*Figure 4: Development of the BC/Al ratio over time in soils of different soil depths and base saturation levels. Error bars: 95% confidence interval, extracted from the mixed regression models (Table S5).*

*Figure 5 has been changed:*

*Figure 5: Changes of BC/Al ratio within five years in relation to selected predictors from Table 3. Predicted values including 95% credible intervals are conditioned on all other fixed effects. Negative changes signify an expected decrease in BC/Al. The linearity of the relations were tested using polynomial functions.*

*Figure 8 has been changed:*

*Figure 9: Comparison of model predictions for the trend of N leaching over time. The complete model ("whole model") included all predictors given in Table 4. The "drought" model included predictions for precipitation (leaching water) and temperature (potential evapotranspiration, site water balance). The "N deposition" model predicted values for N deposition. The model "observed" included the raw data of N leaching. The points are annual means with a loess smoother (degree of smoothing α=0.8) as lines.*

*Reference List*

*1.	Prenzel J (1985) Verlauf und Ursachen der Bodenversauerung. Zeitschrift der deutschen geologischen Gesellschaft 136: 293-302.*

*2.	Ulrich B (1987) Stability, elasticity and resistance of terrestrial ecosystems with respect to matter balance. Ecological Studies 61: 11-49.*

*3.	Ulrich B, Mayer R, Matzner E (1986) Vorräte und Flüsse der chemischen Elemente. In: Ellenberg H, Mayer R, Schauermann J, editors. Ökosystemforschung - Ergebnisse des Solling-Projekts. Stuttgart: Eugen Ulmer GmbH & Co.*

*4.	Falkengren-Grerup U (1987) Long-term changes in pH of forest soils in southern Sweden. Environ Pollut 43: 79-90.*

*5.	Thimonier A, Dupouey JL, Bost F, Becker M (1994) Simultaneous eutrophication and acidification of a forest ecosystem in North-East France. New Phytol 126: 533-539.*

*6.	Högberg P, Näsholm T, Franklin O, Högberg MN (2017) Tamm Review: On the nature of the nitrogen limitation to plant growth in Fennoscandian boreal forests. For Ecol Manage 403: 161-185.*

*7.	Maas, R. and Grennfelt, P. (2016) Towards Cleaner Air. Scientific Assessment Report 2016. EMEP Steering Body and Working Group on Effects of the Convention on Long-Range Transboundary Air Pollution.*

*8.	Meesenburg H, Ahrends B, Fleck S, Wagner M, Fortmann H, Scheler B, Klinck U, Dammann I, Eichhorn J, Mindrup M, Meiwes KJ (2016) Long-term changes of ecosystem services at Solling, Germany: Recovery from acidification, but increasing nitrogen saturation? Ecological Indicators 65: 103-112.*

*9.	Braun S, Cantaluppi L, Flückiger W (2005) Fine roots in stands of Fagus sylvatica and Picea abies along a gradient of soil acidification. Environ Pollut 137: 574-579.*

*10.	Braun S, Schindler C, Volz R, Flückiger W (2003) Forest damage by the storm "Lothar" in permanent observation plots in Switzerland: the significance of soil acidification and nitrogen deposition. Water Air and Soil Pollution 142: 327-340.*

*11.	Braun S, Schindler C, Rihm B (2020) Foliar nutrient concentrations of European beech Switzerland: relations with nitrogen deposition, ozone, climate and soil chemistry. Frontiers in Forests and Global Change .*

*12.	Braun, S. (2018) Untersuchungen über die Zusammensetzung der Bodenlösung. Bericht 2017.* [*http://www.bafu.admin.ch/wald:*](http://www.bafu.admin.ch/wald:) *Institut für Angewandte Pflanzenbiologie. 123 p.*

*13.	Thimonier A, Kosonen Z, Braun S, Rihm B, Schleppi P, Schmitt M, Waldner P, Thöni L (2018) Total depositition of nitrogen in Swiss forests: comparison of assessment methods and evaluation of changes over two decades. Atmospheric Environment 198: 335-350.*

*14.	Pannatier EG, Thimonier A, Schmitt M, Walthert L, Waldner P (2011) A decade of monitoring at Swiss Long-Term Forest Ecosystem Research (LWF) sites: can we observe trends in atmospheric acid deposition and in soil solution acidity? Environmental Monitoring and Assessment 174: 3-30.*

*15.	Emmett B (2007) Nitrogen saturation of terrestrial ecosystems: some recent findings and their implications for our conceptual framework. Water Air and Soil Pollution: Focus 7: 99-109.*

*16.	Lovett G, Goodale C (2011) A new conceptual model of nitrogen saturation based on experimental nitrogen addition to an oak forest. Ecosystems 14: 615-631.*

*17.	Rihm B, Kurz D (2008) Input preparation for dynamic modeling with ForSAFE-VEG in Switzerland - depositon of nitrogen, sulfur and base cations and climate related parameters. In: Sverdrup H, editors. Toward critical loads for nitrogen based on biodiversity. Exploring different methods and first field tests. Background document for the 18th CCE workshop on the assessment of nitrogen effects under the ICP for Modelling and Mapping. Berne: UN-ECE/LRTAP. pp. 38-51.*

*18.	Barbieri A, Pozzi S (2001) Acidifying deposition, Southern Switzerland. -113.*

*19.	Braun, S., Hopf, S., and De Witte, L. (2018) Wie geht es unserem Wald? 34 Jahre Jahre Walddauerbeobachtung. Schönenbuch: Institut für Angewandte Pflanzenbiologie.*

*20.	CLRTAP (2017) Mapping Critical Loads for Ecosytems. Chapter V of Manual on methodologies and criteria for modelling and mapping critical loads and levels and air pollution effects, risks and trends. Update 2017-09-10. UNECE Convention on Long-range Transboundary Air Pollution.*

*21.	Block, J., Eichborn, J., Gehrmann, J., Kölling, C., Matzner, E., Meiwes, K. J., von Wilpert, K., and Wolff, B. (2000) Kennwerte zur Charakterisierung des ökochemischen Bodenzustandes und des Gefährdungspotentials durch Bodenversauerung und Stickstoffsättigung an Level II - Waldökosystem-Dauerbeobachtungsflächen. Bonn: Bundesministerium für Ernährung, Landwirtschaft und Forsten. 167 p.*
